# Sulforaphane Protects Cells against Lipopolysaccharide-Stimulated Inflammation in Murine Macrophages

**DOI:** 10.3390/antiox8120577

**Published:** 2019-11-21

**Authors:** Ruheea Taskin Ruhee, Sihui Ma, Katsuhiko Suzuki

**Affiliations:** 1 Graduate School of Sport Sciences, Waseda University, Tokorozawa 359-1192, Japan; ruhee@fuji.waseda.jp; 2 Faculty of Sport Sciences, Waseda University, Tokorozawa 359-1192, Japan

**Keywords:** sulforaphane, macrophages, inflammation, lipopolysaccharide, cytokines, Nrf2

## Abstract

Inflammation is an essential part for the general or innate immune defenses to defend against tissue damage and accelerate the curing process by providing protection against pathogens. Sulforaphane (SFN) is a natural isothiocyanate that has potential properties against inflammation, along with other protective functions. The purpose of this study was to examine the mechanism of its protective effect on lipopolysaccharide (LPS)-induced inflammation in Raw 264.7 macrophages. Here, we compared LPS-challenged macrophages with or without SFN pretreatment. Macrophages were pre-incubated for 6 h with a wide range of concentrations of SFN (0 to 50 µM), and then treated with LPS for 24 h. Nitric oxide (NO) concentration and gene expression of different inflammatory mediators, i.e., interleukin (IL)-6, tumor necrosis factor (TNF)-α, and IL-1β, were measured. SFN neither directly reacted with cytokines, nor with NO. To understand the mechanisms, we performed analyses of the expression of regulatory enzyme inducible nitic oxide synthase (iNOS), the transcription factor NF-E2-related factor 2 (Nrf2), and its enzyme heme-oxygenase (HO)-1. Our results revealed that LPS increased significantly the expression of inflammatory cytokines and concentration of NO in non-treated cells. SFN was able to prevent the expression of NO and cytokines through regulating inflammatory enzyme iNOS and activation of Nrf2/HO-1 signal transduction pathway.

## 1. Introduction

Plant-derived bioactive compounds are secondary metabolites and are widely known for medicinal use in diseased conditions. These compounds are also known as phytochemicals or phytoprotectants and can influence biochemical reactions inside the cells. Several past researches demonstrated the unique properties of phytonutrient, like anti-aging, free-radical scavenging and cancer chemoprotective activities [1], anti-inflammatory effect and cardioprotective effect, or ability to control LDL (low density lipoproteins) oxidation [2]. Some extended studies found that such bioactive compounds have potential capacity in energy and lipid metabolism, and in reducing the risk of pathologies associated with obesity [3]. Plants are an enriched source of bioactive compounds that have potential biological activity in the human body in the development or prevention of various diseases. Beside some well-known bioactive compounds like polyphenols, flavonoids, etc., a class of organosulfur compounds (OSCs) are also engaged potentially and has been extensively studied. The most studied OSCs from Allium species were garlic and onion, which are familiar because of their biological effects and identical properties [4,5,6]. Vegetables from the Brassicaceae family, also known as cruciferous vegetables (broccoli, cauliflower, cabbage and brussel sprouts) are naturally enriched with glucosinolates (GSTs), which are precursors of isothiocyanaetes, and are of special interest because of the considerable effects on human health [7]. GSTs undergoes enzymatic hydrolysis from its native form by the endogenous plant enzyme myrosinase during cooking or chewing and converted into isothiocyanates, indoles, thiocyanates, and others [8].

**Figure 1:**
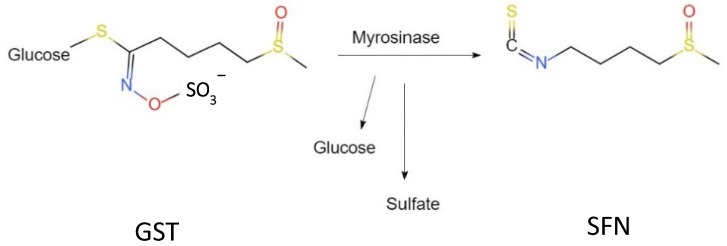
Chemical reaction of SFN and structure.

Sulforaphane (SFN; 1-isothiocyanate-(4R)-(methylsulfinyl) butane; CH_3_S(O)(CH_2_)_4_—N=C=S), is a hydrolysis product of glucoraphanin, belonging to cruciferous vegetables, is one of the most important natural dietary isothiocyanates, and exerts protective effects on multiple organs to protect human health [9]. The synthesis route and chemical structure of SFN are shown in Figure 1. Brussel sprout is considered as the highest source of SFN (1153 mg/100 g dry weight) [10]. Furthermore, the bioavailability of SFN from raw broccoli is 11 times more than the cooked or steamed one [11,12]. It is an indirect antioxidant, which inhibits phase I enzymes like cytochrome P450 and can induce certain phase II enzymes to detoxify reactive oxygen species (ROS) and maintain a balanced condition with oxidants [13,14]. Phase II detoxification enzymes gained special attention because of their xenobiotic mechanism through conjugation reaction. SFN is extensively studied because of its diverse protective effect and/or activity at the transcriptional level. Former studies described that, in diseased conditions, SFN naturally interacts with the transcription factor NF-E2-related factor 2 (Nrf2), which is suppressed in the cytoplasm by the repressor protein Keap1 (Kelch-like erythroid cell-derived protein with cap ‘n’ collar homology-associated protein 1) under homeostatic conditions [15,16]. Upon stimulation, there is a conformational change in the cysteine residues within Keap1, and Nrf2 translocates into the nucleus, followed by interact with antioxidant response element (ARE) [17,18,19]. SFN triggers the reaction promptly by interacting with Nrf2–Keap1 complex, therefore, activating ARE-driven genes, including phase 2 chemoprotective enzymes, i.e., heme oxygenase-1 (HO-1), NAD(P)H: quinoine oxidoreductase 1, glutathione reductase (GR), and glutathione peroxidase (GSH-Px) [20].

Scientific evidence indicates that chronic inflammation is a common phenomenon that causes progressive and irreversible damage in our cellular system [21], because there is an overflow of inflammatory mediators like cytokines and chemokines in the inflamed site which released into the circulation [22]. Some cytokines, especially interleukin (IL)-6, tumor necrosis factor (TNF)-α, and IL-1β are actively produced in large numbers from macrophages in response to inflammation [23]. Furthermore, nitric oxide (NO), another hallmark of inflammation, is catalyzed by the pro-inflammatory enzyme inducible nitric oxide synthase (iNOS), which is further upregulated by the secretion of cytokines in immune cells [24]. However, inhibition of overexpressed inflammatory response could be a possible way to reduce the damage caused by the excessive inflammatory response [25]. In this study we investigated the protective effects of SFN on downregulation of inflammatory cytokines in lipopolysaccharide (LPS)-stimulated macrophages. In addition to that, we also discussed the probable mechanism of anti-inflammatory effect of SFN through upregulation of Nrf2/HO-1 gene expression. 

## 2. Materials and Methods

### 2.1. Cell Culture

Raw 264.7 murine macrophages (DS Pharma Biomedical, Osaka, Japan) were cultured in high glucose DMEM (Dulbecco’s Modified Eagle Medium; Thermo Fisher, Rockford, IL, USA, REF 11995-065, LOT 2045124), with 10% fetal bovine serum (FBS; Biowest Ltd., Loire Valley, France), 100 U/mL penicillin, and 100 µg/mL streptomycin (Thermo Fisher, Rockford, IL, USA), under endotoxin free, at 37 °C with 5% CO_2_ atmosphere. To maintain cell growth, cell culture medium was changed every 2–3 days and continued until 70% confluence. Before seeding into plates, cell suspension was made by frequent pipetting, and percent of viable cells was calculated using trypan blue dye exclusion assay (Thermo Fisher, Rockford, IL, USA, REF 15250061, LOT 2042440).

### 2.2. Cytotoxicity Assay

To perform cytotoxicity assay, the Cytotoxicity LDH (lactate dehydrogenase) assay kit-WST (Dojindo Molecular Technologies, Inc., City, Japan) was used according to manufacturer’s protocol. Cells were seeded in a 96-well plate at a concentration of 3 × 10^5^ cells/mL, kept for 24 h with serum-free DMEM medium. Then, pre-treated with various concentrations of SFN (0 to 50 µM, SFN was purchased from LKT Laboratories, St. Paul, USA, product code S8044) for 6 h, followed by added LPS (1 µg/mL, from *Escherichia coli* 055: B5, purchased from Sigma St. Louis, MO, USA, LOT 074M4052V) and kept for 24 h. A wide range of SFN concentrations were selected to ensure the safest concentration and the LPS concentration were chosen based on the previous experiments to maintain a standardized procedure [26]. Cell culture media were collected, then added working solution, kept in the dark for 30 min. Absorbance was measured at 490 nm by a microplate reader (VERSA max, tunable microplate reader, California, USA). Cytotoxicity is expressed as a percentage of total amount of LDH released from lysed cells. The experiment was performed using three independent samples in each group.

### 2.3. Nitrite Assay 

After treatment with SFN and LPS, nitrite was measured in cell culture supernatant to assess nitric oxide (NO) production, using a Nitrate/Nitrite Colorimetric Assay Kit (Cayman Chemical Co., Ann Arbor, MI, USA) according to manufacturer’s protocol. Briefly, in a 96-well plate, 100 µL of cultured supernatant was mixed with Griess reagent R1 and R2 and incubated for 10 min at room temperature. Absorbance was measured at 540 nm by a microplate reader (VERSA max, tunable microplate reader, California, USA). Nitrite concentration in each sample was calculated using nitrite standard curve. The experiment was performed using three independent samples in each group.

### 2.4. Real Time (RT) Quantitative Polymerase Chain Reaction (qPCR)

Cells were seeded in 6-well plates and cultured for 24 h in serum-free medium. After treatment with SFN for 6 h, followed by LPS for 24 h, total RNA was extracted using TRizol (Thermo Fisher, Rockford, IL, USA) extraction reagent, according to the protocol. NanoDrop system (NanoDrop Technologies, Wilmington, DE, USA) used to confirm the concentration and purity of extracted RNA by the ration of A260/280. Total RNA was reverse transcribed to cDNA using the High-Capacity cDNA Reverse Transcription Kit (Applied Biosystems, Foster City, CA, USA) according to the provided instructions. PCR was performed using the Fast 7500 real-time PCR system (Applied Biosystems, Foster City, CA, USA) and Fast SYBR Green PCR Master Mix (Applied Biosystems, Foster City, CA, USA). Thermal profile for all genes consisted of one denaturing cycle at 95 °C for 10 min, 40 cycles consisting of denaturing at 95 °C for 3 s and annealing and elongation at 60 °C for 15 min. *β-actin* mRNA was used as housekeeping gene. All data were normalized using the ΔΔCT method and stated as a fold change relative to the values of the control group. The sequences of primers used for gene amplification are given in Table 1. Experiments were performed using three independent samples in each group.

### 2.5. Statistical Analysis

All experiments were performed with three independent samples for each group. Data analysis was performed using IBM SPSS v25. Data are expressed as mean ± standard error (SE) and analyzed using Kruskal–Wallis with Dunn’s post-hoc test. Significant differences were set at *p* < 0.05.

## 3. Results

### 3.1. Effect of SFN Pre-Treatment on LPS-Stimulated Cytotoxicity

**Figure 2:**
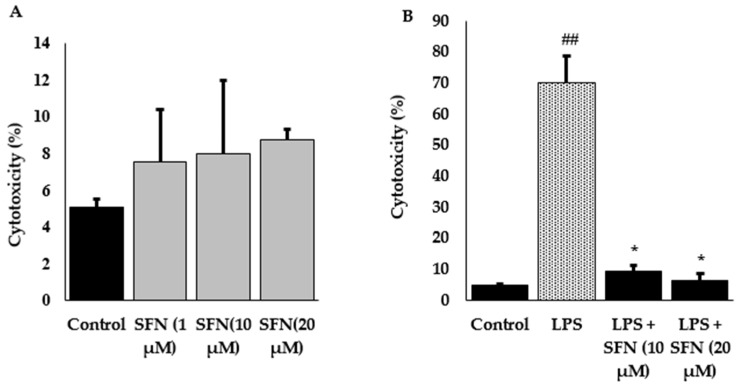
Raw 264.7 cells were exposed to various concentrations of SFN and the cytotoxicity was measured as % of LDH released into the culture supernatant. The relative LDH release is calculated by the ratio of LDH release over control sample. Different concentrations of SFN (0–20 µM) treated in raw 264.7 cells showed non-significant results compare to the control (**A**). On the other hand, pre-treatment with SFN for 6 h, then administration of LPS for 24 h showed a significant reduction of LDH release as compared to LPS alone (**B**). The results are expressed as mean ± standard error (SE) (## *p* < 0.01 compared to control; * *p* < 0.01 compared to LPS only).

To avoid the toxic effect of SFN, cell cytotoxicity test was performed. Cells were incubated with different concentrations of SFN (up to 50 µM) or LPS alone, or combined with SFN; however, SFN concentration up to 20 µM did not show any significant differences in cytotoxicity (Figure 2A). Besides, LPS-stimulated cells showed a higher percentage of cytotoxicity, whereas SFN pre-treated cells showed significantly reduced LPS-stimulated cytotoxicity, compare to untreated cells (Figure 2B).

### 3.2. Effect of SFN on Nitrite Production and iNOS Expression

**Figure 3:**
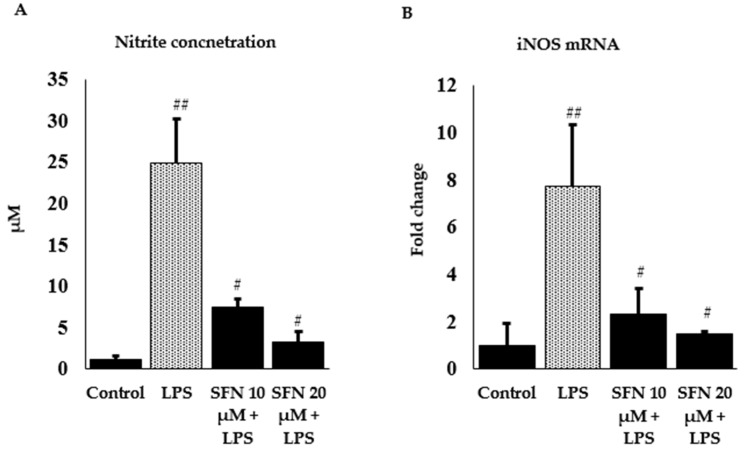
Effect of SFN on LPS induced nitrite concentration and *iNOS* mRNA expression. (**A**) SFN pre-treatment reduced LPS induced nitrite concentration (µM) in raw 264.7 cells, y-axis represents concentration and (**B**) mRNA expression of *iNOS*. Raw 264.7 cells were incubated with SFN (10 and 20 µM) for 6 h, then incubated with LPS in both SFN pre-treated and untreated cells for 24 h. Fold changes are presented after normalization to the internal control *β-actin*. The results are expressed as mean ± SE (## *p* < 0.01 compared to control; # *p* < 0.05 compared to LPS only).

Nitrite is a stable oxidized product of NO, which is an important biological mediator, catalyzed by iNOS and produced at a higher concentration during inflammation or injury. In our experiment, we measured the protective effect of SFN against LPS-induced nitrite level and *iNOS* mRNA expression in SFN pre-treated cells using Griess reagent and RT-qPCR. LPS-stimulation caused a significant increase in nitrite concentration around 68%, whereas SFN-treated cells (10 and 20 µM) significantly showed decreased concentrations (Figure 3A) by 24% and 9%, accordingly, comparing with the control. Furthermore, LPS treatment increased *iNOS* gene expression by 7.7 -fold, whereas SFN (20 µM) potentially downregulated *iNOS* mRNA expression by 1.5-fold (Figure 3B).

### 3.3. Effect of SFN on Inflammatory Cytokines and Enzyme Expression through Nrf2/HO-1 Signaling Pathway

**Figure 4:**
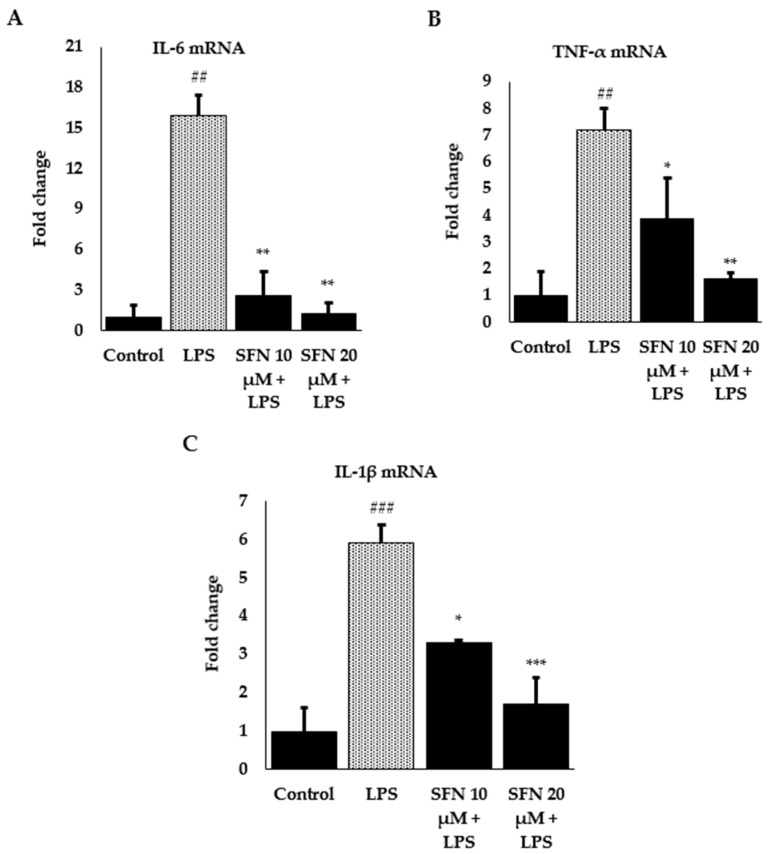
Effect of SFN on LPS-stimulated inflammatory cytokine expression. (**A**) *IL-6* mRNA expression, (**B**) *TNF-α* mRNA expression, **(C)** *IL-1β* mRNA expression. Raw 264.7 cells were incubated with SFN (10, 20 µM) for 6 h, then incubated with LPS in both SFN pre-treated and untreated cells for 24 h. Total RNA was extracted and prepared for mRNA gene expression of *IL-6*, *TNF-α* and *IL-1β* using RT-qPCR. Fold changes are presented after normalization to the internal control *β-actin*. The results are expressed as mean ± SE (### *p* < 0.001, ## *p* < 0.01 as compared to the control; *** *p* < 0.001, ** *p* <0.01, * *p* < 0.05 as compared to LPS only).

To investigate the anti-inflammatory effects and the mechanisms, we further measured mRNA expression of pro-inflammatory cytokines IL-6, TNF-α, and IL-1β by RT-qPCR. Gene expression in the unstimulated cells was hard to detect; however, LPS stimulation markedly increased *IL-6*, *TNF-α*, and *IL-1β* expression by 15.9, 7.2, and 7.2 -fold, respectively, which was reduced by SFN pre-treatment at different concentrations (Figure 4).

**Figure 5:**
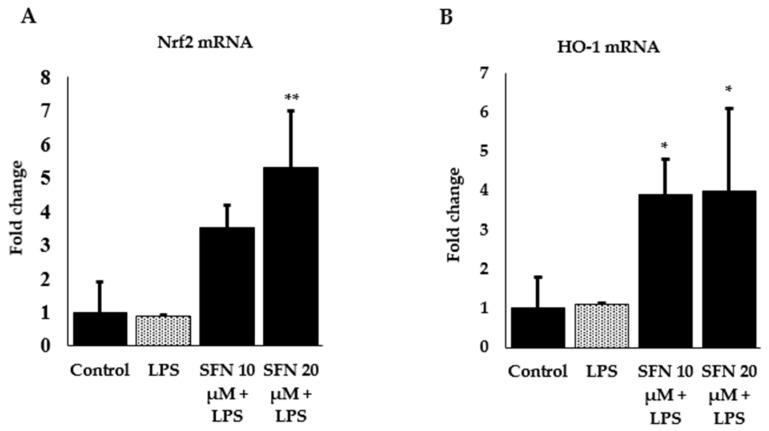
Effect of SFN on upregulation of transcription factor Nrf2 (**A**) and phase 2 enzyme HO-1 (**B**) in LPS-stimulated cells. Raw 264.7 cells were incubated with SFN (10 and 20 µM) for 6 h, then incubated with LPS in both SFN pre-treated and untreated cells for 24 h. Total RNA was extracted and prepared for mRNA gene expression of *Nrf2* and *HO-1* by RT-qPCR. Fold changes are presented after normalization to the internal control *β-actin*. The results are expressed as mean ± SE (** *p* < 0.01, * *p* < 0.05 as compared to LPS only).

We further measured gene expression of transcription factor Nrf2 and phase 2 enzyme HO-1 to identify the underlying mechanisms involved in the protection against inflammation by SFN in LPS-stimulated macrophages. As shown in Figure 5A *Nrf2* mRNA expression significantly upregulated in the SFN (20 µM)-treated cells, whereas extremely low amplification was found for LPS stimulation alone. A similar effect was observed for *HO-1* (Figure 5B), which indicates that the anti-inflammatory effects of SFN may be attributed to the downregulation of pro-inflammatory cytokines at the transcriptional level by activating Nrf2 and with the induction of HO-1.

## 4. Discussion

In this study, we presented the potential protective effect of SFN in LPS-induced murine macrophage cells. SFN is considered as a very potent bioactive compound obtained from cruciferous vegetables. During inflammation, macrophages play a vital role in immunomodulation through regulation of cytokine production and other mediators. When any foreign particles enter inside our body, activated macrophages release inflammatory cytokines and enzymes. On the contrary, anti-inflammatory cytokines produced by macrophages reduce the inflammatory cytokine expression. Thereby, throughout the inflammatory process of initiation and prevention, macrophages have dual roles [27]. LPS is an endotoxin, released from the outer membrane of Gram-negative bacteria, to mimic the state of bacterial invasion as an original manifestation of bacterial infection. Generally, the immune system lyses the bacteria and releases the lipid part of LPS into the circulation, which initiates endotoxic activity and triggers the release of pro-inflammatory mediators from macrophages [28,29]. Past experiments revealed that LPS-challenged macrophages excessively produced TNF-α and IL-6, along with other cytokines [30,31]. In order to suppress the activation of macrophages, adequate antioxidant supply is needed through the diet, which may further repair the damaged site [32].

Nrf2 regulates expression of numerous genes, and it has a very important role in the regulation of inflammation, besides providing protection against oxidative stress. It is well established that activation of Nrf2 provides an effective protection from cancer [33], diabetic nephropathy [34], and chronic liver diseases [35] by upregulating ARE-related detoxifying enzymes [36]. NF-κB (Nuclear Factor kappa-light-chain-enhancer of activated B cells), another pivotal transcriptional factor in our immune system, is readily activated in an oxidative environment and regulates expression of inflammatory genes [37]. Wardyn et al. summarized the coordinated cellular responses and cross-talks of Nrf2 and NF-κB [38]. However, depletion of Nrf2 causes an augmentation of NF-κB activity and inflammatory responses [39]. On the contrary, NF-κB can suppress Nrf2 activation through an interaction with CREB binding protein (CBP) [40]. Further definite evidences are required for revealing the interplay between Nrf2 and NF-κB.

**Figure 6:**
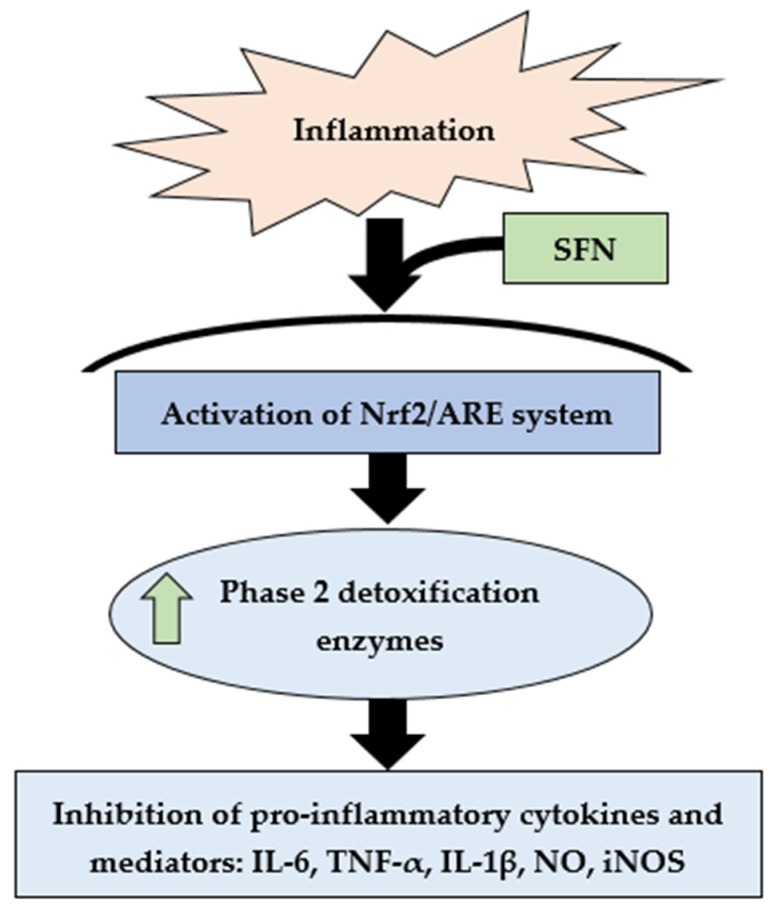
Summary of SFN-mediated activation of Nrf2/ARE-driven phase 2 detoxifying enzyme expression and potential inhibition of pro-inflammatory cytokines and other mediators.

Several murine models evidenced that Nrf2 deficiency can increase inflammation, as well as cytokines and regulatory enzymes [41,42]. Peritoneal neutrophils collected from Nrf2 knockout (nrf2 −/−) mice showed an increased expression of cytokines (IL-6, TNF-α) after LPS stimulation, as compared to wild-type (nrf2 +/+) neutrophils [43]. SFN is a very important phytochemical that can activate Nrf2; therefore, it could inhibit the progression of inflammation [42]. SFN has also been involved in protecting against carcinogenic stimuli via epigenetic modulation [44] against diabetes-induced cardiac damage, along with a range of cardiovascular diseases with prolonged supplementation [45]. It can modify the sulfhydryl group, oxidize cysteine residues in the Keap1–Nrf2 complex, and release Nrf2 into the nucleus [46]. Attenuation of inflammatory cytokines, NO, and iNOS is considered as a useful approach to protect against chronic diseases [47]. Here, we showed that (Figure 6) SFN treatment may reduce mRNA expression of inflammatory cytokines and mediators *(IL-6*, *TNF-α*, *IL-1β*, *NO*, *iNOS*) via upregulation of *Nrf2* mRNA expression. 

HO-1 is one of two HO isoforms, an Nrf2 target gene, expressed by Nrf2 activation [48]. HO catalyzes the rate limiting step in heme degradation and produces free iron, biliverdin, and carbon monoxide. Some important biological processes like inflammation, apoptosis, fibrosis, and angiogenesis are actively regulated by HO-1 [49,50]. Here, SFN further increased expression of HO-1 enzyme and suppressed the expression of pro-inflammatory cytokines and iNOS and NO significantly. This observation may describe the protective effect of SFN against inflammation. Previous studies described that Nrf2/HO-1 signaling pathway could be a promising strategy in preventing neuroinflammation [51] and gastrointestinal tract inflammation [52], and in exerting vital roles in the treatment of a variety of diseases [53].

NO is an important biological mediator in the immune system, synthesized from L-arginine by the enzymes of nitric oxide syntheses (NOS), mainly inducible NOS (iNOS) and endothelial NOS (eNOS) [54]. LPS stimulation produces inflammatory cytokines from activated macrophages that further culminates iNOS expression, which oxidizes L-arginine and releases NO [55]. Pre-treatment with SFN can reduce NO production in a concentration-dependent manner [56]. Our study pointed on a 6-h pre-incubation period with different concentrations of SFN, followed by LPS stimulation for 24 h, which suppressed the expression of inflammatory cytokine expression and regulatory enzyme (iNOS), and as a consequence, found reduced NO release from macrophages.

It has been demonstrated that the anti-inflammatory effect of SFN depends on Nrf2 activation. Furthermore, SFN at a lower concentration (10 or 20 µM) prevented inflammatory cytokine expression, which can be readily obtained by daily consumption of 100–200 g broccoli sprouts [12]. In this study we used a concentrated dose of LPS to mimic severe sepsis [26] and focused on the potential anti-inflammatory effect and protective mechanism of SFN.

## 5. Conclusions

Based on the findings, we demonstrated conclusively that Nrf2 signaling appears to be an important mechanism in the upregulation of phase 2 enzymes and downregulation of inflammatory cytokines, including inflammatory enzymes. Protection against inflammation can be conferred by activating Nrf2-mediated antioxidant response element pathway by using SFN. Generally, fruits and vegetables comprise bioactive compounds which possess diverse health benefits, besides that they are comparatively inexpensive, non-toxic and ensure healthy diet. Brassica-derived isothiocyanate SFN is such an important phytochemical, which possesses numerous protective effects. In current research fields, SFN is drawing special attention due to its promising positive strategy towards human health. Since this study only discussed the gene expression of inflammatory cytokines and mediators, further studies are required in order to identify a more definitive explanation of the promising effects of SFN. To extrapolate the findings, currently investigating the anti-inflammatory effect of SFN in a mouse model through oral administration.

## Figures and Tables

**Table 1:** Specific primers sequence for RT-qPCR.

Target Gene	Accession Number	Forward	Reverse
*β-actin*	NM_007393	5′-GCGGACTGTTACTGAGCTGCGT-3′	5′-TGCTGTCGCCTTCACCGTTCC-3′
*IL-6*	NM_031168	5′-TAGTCCTTCCTACCCCAATTTCC-3′	5′-TTGGTCCTTAGCCACTCCTTC-3′
*TNF-α*	NM_013693	5′-TCTTCTCATTCCTGCTTGTGG-3′	5′-GAGGCCATTTGGGAACTTCT-3′
*IL-1β*	NM_008361.4	5′-GGGCCTCAAAGGAAAGAATC-3′	5′-TTCCAGAATCCCTGGACAAG-3′
*iNOS*	NM_010927.3	5′-GCAAACCCAAGGTCTACGTTCA-3′	5′-GAGCACGCTGAGTACCTCATTG-3′
*Nrf2*	NM_010902	5′-GAGTCGCTTGCCCTGGATATC-3′	5′-TCATGGCTGCCTCCAGAGAA-3′
*HO-1*	NM_010442	5′-CACGCATATACCCGCTACCT-3′	5′-CCAGAGTGTTCATTCGAGCA-3′
